# Higher S100B Levels Predict Persistently Elevated Anhedonia with Escitalopram Monotherapy Versus Antidepressant Combinations: Findings from CO-MED Trial

**DOI:** 10.3390/ph12040184

**Published:** 2019-12-17

**Authors:** Manish K. Jha, Abu Minhajuddin, Bharathi S. Gadad, Cherise Chin Fatt, Madhukar H. Trivedi

**Affiliations:** 1 Department of Psychiatry, Icahn School of Medicine at Mount Sinai, New York, NY 10029, USA; manish.jha@utsouthwestern.edu; 2 Center for Depression Research and Clinical Care, University of Texas Southwestern Medical Center, Dallas, TX 75235, USA; abu.minhajuddin@utsouthwestern.edu (A.M.); Bharathi.Gadad@utsouthwestern.edu (B.S.G.); cherise.chinfatt@utsouthwestern.edu (C.C.F.); 3 Department of Psychiatry, Texas Tech University Health Science Center, El Paso, TX 79905, USA

**Keywords:** S100B, anhedonia, antidepressant response, SSRIs, bupropion, blood–brain barrier, moderator, dopamine, serotonin

## Abstract

Background: Elevated S100 calcium binding protein B (S100B) levels in systemic circulation may induce neuroinflammation and reflect greater blood–brain barrier (BBB) dysfunction. Neuroinflammation in patients with major depressive disorder (MDD), in turn, may reduce likelihood of improvement with serotonergic antidepressants. Methods: Levels of S100B were measured in plasma samples obtained prior to initiation of treatment with bupropion-plus-escitalopram, escitalopram-plus-placebo, or venlafaxine-plus-mirtazapine in participants of Combining Medications to Enhance Depression Outcomes trial (*n* = 153). Depression severity was measured with 16-item Quick Inventory of Depressive Symptomatology Self-Report and anhedonia was measured with 3 items of 30-item Inventory of Depressive Symptomatology. Differential changes in depression severity and anhedonia over acute-phase (baseline, weeks 1, 2, 4, 6, 8, 10, and 12) in the three treatment arms were tested with logS100B-by-treatment-arm interaction in mixed model analyses after controlling for age, gender, and body mass index. Results: There was a significant logS100B-by-treatment-arm interaction for anhedonia (F = 3.21; df = 2, 142; *p* = 0.04) but not for overall depression severity (F = 1.99; df = 2, 142; *p* = 0.14). Higher logS100B levels were associated with smaller reductions in anhedonia (effect size = 0.67, *p* = 0.047) in escitalopram monotherapy but not in the other two arms. Correlation coefficients of anhedonia severity averaged over acute-phase (including baseline) with baseline S100B levels were 0.57, −0.19, and 0.22 for escitalopram monotherapy, bupropion-plus-escitalopram and venlafaxine-plus-mirtazapine arms respectively. Conclusion: Higher baseline S100B levels in depressed patients resulted in poorer response to escitalopram monotherapy. Addition of bupropion, a dopaminergic antidepressant, partially mitigated this effect.

## 1. Introduction

Stress can increase levels of pro-inflammatory cytokines in peripheral circulation as well as in central nervous system (CNS) due to increased permeability of blood–brain barrier (BBB) [1,2]. Enhancement of BBB integrity, in turn, may mitigate the effect of acute stress in animal models and has been shown to reduce the rate of development of learned helplessness and lower levels of circulating inflammatory cytokines [2]. Dysfunction of BBB may partly explain the CNS effects of peripheral inflammation [3]. In a recent report, levels of c-reactive protein (CRP), a non-specific marker of inflammation synthesized by liver, in blood and in cerebrospinal fluid (CSF) were highly correlated [4]. Further, elevated levels of CRP in this report were associated with increased levels of inflammatory cytokines in CSF [4]. Taken together, these findings suggest propagation of peripheral immune signals to central nervous system (CNS) which is reflected in reports linking peripheral inflammation to impaired reward behaviors and symptoms of anhedonia [5,6]. Thus, an improved understanding of how inflammation relates to BBB dysfunction may have immediate application in developing novel antidepressants and in antidepressant treatment selection, both of which continue to be a trial-and-error process [7,8,9]. Recent reports suggest that elevated markers of inflammation such as CRP and interleukin 17 (IL-17) may predict poor outcomes with selective serotonin reuptake inhibitor (SSRI) antidepressants [10,11,12,13,14,15,16]. These findings are consistent with reports linking obesity, a frequent cause of systemic inflammation, to poorer outcomes to SSRIs like escitalopram [17,18]. This report builds on these previous findings of peripheral immune markers and evaluates the prognostic utility of S100 calcium binding protein B (S100B), an intracellular protein expressed mostly in glial cells and a potential peripheral marker of both immune activation and disruption of BBB integrity, in predicting response to acute-phase antidepressant treatment with SSRI monotherapy vs. antidepressant combinations.

Multiple lines of investigation suggest the utility of measuring levels of S100B in depression. At low concentration, S100B in brain serves as a neurotrophic factor. However, higher extracellular levels may indicate reduced BBB integrity and may provoke inflammatory response by engaging receptors for damage associated molecular patterns [19]. Additionally, due to predominantly glial origin, elevated levels of S100B in plasma may also be a marker of reduced integrity of BBB [20,21]. Reduced BBB integrity may be a putative mechanistic link between peripheral inflammation and CNS effects as previous reports suggest that pro-inflammatory cytokines in peripheral circulation bind to the endothelial cells of BBB and result in formation of reactive oxygen species [22]. This resulting oxidative stress has been shown to increase BBB permeability and to facilitate CNS infiltration of peripheral immune cells [23]. Elevated stress and peripheral inflammation have also been shown to damage the BBB and facilitate monocytic infiltration of CNS [24]. As reviewed in detail by Miller et al. [25], inflammatory changes within CNS may result in induction of nitric oxide synthase (NOS) which in turn may reduce synthesis of dopamine by diversion of tetrahydrobiopterin, an essential cofactor of NOS and tyrosine hydroxylase, away from rate limiting step (conversion of tyrosine to L-3,4-dihydroxyphenylalanine) in dopamine synthesis. Thus, assessing BBB integrity with peripheral markers such as S100B may elucidate the biological underpinning of reward dysfunction, including symptoms of anhedonia, that is associated with peripheral inflammation [26].

Several reports have found elevated levels of S100B in peripheral circulation of patients with depression [27,28,29]. Two previous reports have evaluated the association between pre-treatment S100B levels and response to treatment outcomes and found that elevated levels of S100B were associated with better outcomes to antidepressants [30,31]. However, neither of these reports evaluated whether levels of S100B differentially predicted improvement with one antidepressant medication versus another.

In this report, using a sample of convenience from the Combining Medications to Enhance Depression Outcomes (CO-MED) trial, we evaluated if pre-treatment S100B levels differentially predicted response to escitalopram monotherapy versus combinations of escitalopram plus bupropion or venlafaxine plus mirtazapine. We measured changes both in overall depressive symptoms and in anhedonia as we have previously found that inflammation was more strongly linked to anhedonia than other depressive symptoms in CO-MED trial [5]. Thus, in this report, we asked the following specific questions:

(1). Does baseline S100B differentially predict changes in anhedonia with escitalopram monotherapy versus antidepressant combinations?

(2). Does baseline S100B differentially predict changes in overall depression severity with escitalopram monotherapy versus antidepressant combinations?

## 2. Methods

### 2.1. Study Overview

Data for this report were obtained from the CO-MED trial where participants (*n* = 665) were randomized after stratification for site to one of the following treatment arms: escitalopram plus placebo, bupropion sustained-release (SR) plus escitalopram, and venlafaxine extended-release (XR) plus mirtazapine [32]. The analytic sample of this report (*n* = 153) includes a sub-set of CO-MED trial participants who provided plasma samples at baseline as part of a separate add-on optional biomarker study that required an additional consent. As previously reported, participants who did not provide plasma samples in CO-MED trial were younger and had lower use of statin medication than those who provided plasma samples at baseline, but did not differ on any other baseline clinical and sociodemographic features [12]. Additionally, as participation in the continuation-phase of CO-MED was censured for those participants with inadequate response [32], only acute-phase visits (baseline and weeks 1, 2, 4, 6, 8, 10, and 12) were included in this report. The CO-MED trial used broad inclusion and exclusion criteria, (fully listed at https://clinicaltrials.gov/ct2/show/NCT00590863) while recruiting from psychiatric and primary care clinics that were chosen to ensure adequate minority representation and a diverse participant group [32]. The trial was reviewed and approved by the Institutional Review Boards at UT Southwestern Medical Center at Dallas, the University of Pittsburgh Data Coordinating Center, each participating regional center, and all relevant clinics. All subjects gave their informed consent for inclusion before they participated in the study. The study was conducted in accordance with the Declaration of Helsinki, and the protocol was approved by the Ethical and Compliance Committee and the Institutional Review Board for Human Subjects Research (IRB Code Number: 112007-032) of UT Southwestern Medical Center at Dallas, Texas. More details about this study are available at the clinical trials.gov site: https://clinicaltrials.gov/ct2/show/NCT00590863. Additionally, de-identified data for this study has been made publicly available by NIMH at https://nda.nih.gov/edit_collection.html?id=2158.

### 2.2. Medications

Participants in all three treatment arms received two types of pills in single blind fashion (study personnel knew of both pill types, but participants knew only the first pill type). Dosage adjustments were made during the first 8 weeks of participation using principles of measurement-based care, with dose increases permitted only if side effects were tolerable and depression severity was not adequately controlled [33]. Dose escalation regime as well as mean doses of medications in each treatment arm have been previously described in detail by Rush et al. [32]. Briefly, participants in the escitalopram monotherapy arm were started on escitalopram at 10 mg/day and placebo was added at week 2 as the second pill type. At the end of 12 weeks, the mean escitalopram dose was 17.6 mg/day and mean placebo dose was 1.4 pills/day. For the bupropion plus escitalopram arm, participants were started on 150 mg of bupropion SR and titrated to 300 mg/day at week 1 and escitalopram 10 mg/day was added as the second pill type at week 2. At the end of 12 weeks, mean bupropion SR dose was 324.0 mg/day and mean escitalopram dose was 14.0 mg/day. Participants in the venlafaxine-mirtazapine treatment arm were started on venlafaxine XR which was titrated from 37.5 mg/day to 150 mg/day at week 1 visit, and mirtazapine 15 mg/day was added at week 2 as the second pill type. At the end of 12 weeks, the mean venlafaxine XR dose was 207.6 mg/day and mean mirtazapine dose was 25.3 mg/day.

### 2.3. Assessments

Participants provided sociodemographic information (age, sex, race, ethnicity, and years of education) at baseline. Height and weight were obtained at baseline to compute BMI. Clinicians completed a structured diagnostic interview to establish current diagnosis of MDD and to obtain the age of onset of first depressive episode [32]. As previously described by Rush et al. [32], presence of anxious features in CO-MED trial was ascribed at baseline if the score of anxiety somatization factor {anxiety, psychic; anxiety, somatic; somatic symptoms, gastrointestinal; somatic symptoms, general; hypochondriasis; and insight) of Hamilton Rating Scale for Depression (HAM-D) was greater than or equal to 7. At baseline and each treatment visit, participants filled out the 16-item Quick Inventory of Depressive Symptomatology-Self-Report (QIDS-SR) and clinicians completed the 30-item Inventory of Depressive Symptomatology Clinician-Rated (IDS-C).

#### 2.3.1. Quick Inventory of Depressive Symptomatology Self-Report (QIDS-SR)

This commonly used scale has 16 items, each of which includes 4 choices that are scored from 0–3. A total score is calculated from nine of these 16 items (consistent with the nine criterion symptom domains of MDD) leading to a range of 0–27 [34]. In previous reports, the reported Cronbach’s α of QIDS-SR has ranged from 0.86 to 0.87 [34,35,36]. The mean (SD) Cronbach’s α of QIDS-SR across visits (weeks 0–12) was 0.782 (0.041). In the CO-MED trial, the QIDS-SR served as the primary depression symptom severity outcome measure.

#### 2.3.2. Inventory of Depressive Symptomatology Clinician Rated (IDS-C)

Of the 30 items of IDS-C (each item has 4 choices which are scored from 0–3), 28 items are summed to generate a total score (range 0–84) which correlates very highly (Pearson’s moment correlation equals 0.95) with HAM-D [36]. In previous reports, the Cronbach’s α of IDS-C has ranged from 0.67 to 0.94 [37,38]. The mean (SD) Cronbach’s α of IDS-C across visits (weeks 0–12) was 0.844 (0.043). Anhedonia, as measured by a subscale of three items of IDS-C (involvement, pleasure/enjoyment (exclude sexual activities), and sexual interest), has been shown to compare favorably to Snaith–Hamilton Pleasure Scale [26,39] and was used as an outcome for this report. The mean (SD) Cronbach’s α of IDS anhedonia subscale across visits (weeks 0–12) was 0.655 (0.038).

### 2.4. Measurement of S100B Levels in Plasma

Plasma samples used in this report (*n* = 153) were obtained from the Biologic Core of National Institute of Mental Health Repository and Genomics Resource (NIMH RGR) and transported to UT Southwestern on dry ice for storage at −80 °C until immediately prior to assays without any freeze/thaw cycles. During CO-MED trial, plasma extracted from participants were transported overnight to the Biologic Core of National Institute of Mental Health Repository and Genomics Resource (NIMH RGR) for storage at −80 °C. Levels of S100B protein were measured in all plasma samples by the Metabolic Phenotyping Core, UT Southwestern Medical Center at Dallas using the Human S100B enzyme-linked immunosorbent assay (ELISA) kit (Human S100 calcium binding protein B ELISA Kit, Catalog No. MBS2503148) from MyBioSource Co, USA using the ELISA Microplate reader (TECAN) according to the manufacturer’s instructions. The concentration gradients of the kit standards or positive controls have a detection range from 31.25–2000 pg/mL with an estimated sensitivity of 18.75 pg/mL. The sandwich ELISA is based on combination of polyclonal antibody coated microtiter plate and HRP-labelled (HRP, horseradish peroxidase) monoclonal antibody conjugate. Three incubation steps were included (1st-standards, samples or controls, 2nd-conjugate, 3rd-substrate) separated by washing steps. Then optical density was measured at 450 nm and the concentration of S100B in pg/mL were calculated using a system-generated four parameter logistic (4-PL) curve-fit for the standards and the samples. The inter- and intra-assay coefficients of variation were 5% and 6.5%, respectively. Levels of other immune markers, including CRP, were available from previously reported analyses [11,12,15].

### 2.5. Statistical Analyses

Baseline sociodemographic and clinical features were compared among the three treatment arms using analysis of variance and chi-square test for continuous and categorical variables respectively. Levels of all biomarkers, including S100B were log transformed due to skewed distribution. Pearson’s’ correlation coefficient were estimated to evaluate the association between S100B and peripheral immune markers. Separate repeated measures mixed model analyses with depression severity (QIDS-SR) and anhedonia (IDS-C anhedonia subscale) were used to evaluate if treatment outcomes differed by baseline S100B levels using a baseline log S100B-by-treatment arm interaction. The symptom measures were used as continuous outcomes in mixed model analyses to maximize the power and utilize all available data. To interpret a significant interaction, analyses were repeated after stratification for treatment arm. Age, gender, and BMI were used as covariates for these mixed model analyses. To evaluate if these findings were significant even after controlling for baseline severity, we repeated these stratified mixed model analyses with anhedonia from week-1 to week-12 as the outcome variable and baseline anhedonia as a covariate. All analyses were conducted with SAS 9.4 version. Threshold for statistical significance was set at *p* < 0.05.

## 3. Results

The three treatment arms did not differ on baseline sociodemographic status except for age which was significantly lower in the venlafaxine plus mirtazapine arm, Table 1. Additionally, levels of S100B did not differ among the three treatment arms at baseline. Levels of S100B were correlated with those of IL-17 (r = 0.20, *p* = 0.013), IL-4 (r = 0.24, *p* = 0.002), IL-5 (r = 0.22, *p* = 0.007), platelet derived growth factor (r = 0.16, *p* = 0.046), IL-6 (r = 0.19, *p* = 0.023), and IL-9 (r = 0.18, *p* = 0.026). There was no significant association of S100B with levels of CRP (r = 0.10, *p* = 0.23).

### 3.1. Does Baseline S100B Differentially Predict Changes in Anhedonia with Escitalopram Monotherapy versus Antidepressant Combinations?

**Figure 1:**
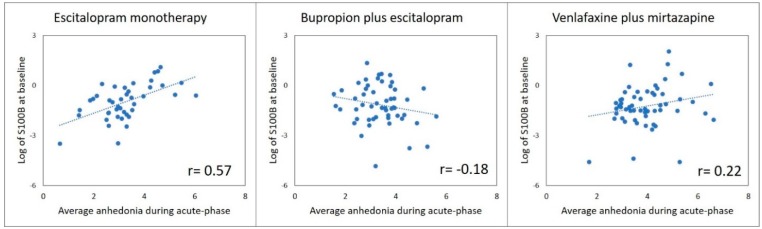
Higher S100B at baseline is associated with persistently elevated anhedonia during acute-phase treatment with escitalopram monotherapy. Legends: S100B is S100 calcium binding protein B.

Yes. There was a significant interaction between baseline S100B level and treatment arm in predicting levels of anhedonia during acute-phase antidepressant treatment (F = 3.21, df = 1, 142, *p* = 0.043) even after controlling for age, gender, and BMI, see Table 2. In subsequent analyses stratified by treatment arm, the effect of baseline S100B was significant in the escitalopram monotherapy arm (F = 4.20, df = 1, 37, *p* = 0.048) but not in bupropion plus escitalopram (F = 1.76, df = 1, 48, *p* = 0.19) or in venlafaxine plus mirtazapine arm (F = 0.81, df = 1, 51, *p* = 0.37). As shown in Figure 1, participants of CO-MED trial with higher levels of S100B at baseline and treated with escitalopram monotherapy experienced smaller reductions in anhedonia with treatment (as reflected in higher anhedonia averaged overall in all visits including baseline). This trend was reversed in the bupropion escitalopram group but not in venlafaxine mirtazapine group. The effect of baseline S100B continued to be significant (F = 4.90, df = 1, 33, *p* = 0.034) in escitalopram monotherapy arm in mixed model analyses with levels of anhedonia from weeks 1–12 as outcome and baseline anhedonia as an additional covariate. Furthermore, in these mixed model analyses, there was no significant effect of baseline S100B on levels of anhedonia in bupropion plus escitalopram ((F = 0.46, df = 1, 45, *p* = 0.499) and venlafaxine plus mirtazapine (F = 0.00, df = 1, 47, *p* = 0.999).

### 3.2. Does Baseline S100B Differentially Predict Changes in Overall Depression Severity with Escitalopram Monotherapy versus Antidepressant Combinations?

**No.** There was no significant baseline S100B-by-treatment arm interaction (F = 1.99, df = 2, 142, *p* = 0.14) for levels of overall depression severity, as measured by QIDS-SR, also see Table 2.

## 4. Discussion

In this report of outpatients with MDD, we found that levels of S100B at baseline predicted persistently elevated levels of anhedonia with escitalopram monotherapy but not with bupropion plus escitalopram or venlafaxine plus mirtazapine. Further, we found that this association was specific to anhedonia as there was no treatment arm by S100B interaction for overall depression severity.

Findings of this report are consistent with previous findings linking elevated levels of inflammatory markers, such as CRP or IL-17 with poorer response to escitalopram monotherapy [11,12]. However, it is noteworthy that levels of S100B were either not correlated with immune markers (such as CRP) or had a significant but small association (such as IL-17). This likely reflects the complexity of immune system and the syndromic nature of MDD. Among the different blood-based immune markers, CRP appears to be the most pragmatic and currently available for clinical use [14], while markers such as S100B may elucidate pathophysiological mechanisms and guide development of novel treatments.

While this is the first report to look at treatment arms separately, our findings that bupropion plus escitalopram combination (resembling in pharmacological action to serotonin norepinephrine reuptake inhibitors) may have trended towards greater efficacy in those with elevated S100B is partly consistent with previous findings of responders to venlafaxine and imipramine (both serotonin norepinephrine reuptake inhibitors) having elevated levels of S100B at baseline [30]. However, our findings differ from those of Jang et al. who found that elevated levels of S100B predicted better outcomes to antidepressants (a mix of various meds but mostly mirtazapine and fluoxetine) as we did not find any association between levels of S100B at baseline and levels of overall depression severity across study visits as measured by QIDS-SR [31].

By emphasizing a stronger link of immune-related markers with anhedonia than overall depressive symptoms, findings of this report argue against the use of overall depressive symptoms as the treatment outcome for studies of anti-inflammatory treatments in patients with MDD [8]. This may be important as in a recently completed study of Sukuma (anti-IL-6 monoclonal antibody) in patients with MDD, while there was no significant difference in overall depression severity between sirukumab and placebo, sirukumab was more effective than placebo in reducing severity of anhedonia [40]. Furthermore, findings of this study also argue for better assessment of BBB integrity with imaging modalities to elucidate the mechanistic link between peripheral inflammation and CNS dysfunction.

There are several limitations of this report. As finding a difference between treatment arms based on pre-treatment S100B levels was not a primary aim of CO-MED, the study may not have been adequately powered to detect these differences. As such, these reports should be considered preliminary. Use of IDS-C to define anhedonia is limited and future studies that assess anhedonia more comprehensively (such as anticipatory, consummatory, and social anhedonia) are needed. Use of plasma samples only to measure S100B and lack of cerebrospinal fluid samples is another limitation. Furthermore, prospective studies are needed to establish the clinical utility of measuring S100B in driving treatment precision. This report is also limited by assessment of S100B at one time-point only and by inclusion of treatment-seeking outpatients. An ongoing large observation study, Texas Resilience Against Depression (T-RAD), offers the opportunity to validate and extend these findings in a large sample of depressed outpatients by using both clinical and neuroimaging data collected over several years [41]. This study is also enrolling youths and young adults at-risk for depression, which may elucidate whether immune changes precede neural circuit dysfunction and syndromic presentation. Finally, novel and comprehensive computational strategies are needed that can combine information across multiple modalities [42] to fully capture the complexities of immune system and how it relates to syndromic manifestation of MDD.

In conclusion, this report identified S100B as a novel blood-based biomarker that predicts differential changes in anhedonia with escitalopram monotherapy versus antidepressant combinations. These findings argue for careful consideration of immune dysfunction and blood–brain barrier integrity in patients with MDD.

## Figures and Tables

**Table 1:** Baseline sociodemographic and clinical characteristics of Combining Medications to Enhance Depression Outcomes (CO-MED) trial participants (*n* = 153) who provided plasma at baseline.

	Total		Escitalopram Monotherapy		Bupropion Plus Escitalopram		Venlafaxine Plus Mirtazapine			*p*-Value
Number	153		44		53		56			
Categorical variables	N	%	N	%	N	%	N	%	χ^2^ (df)	
Sex									0.03 (2)	0.98
Male	45	29.4	13	29.6	16	30.2	16	28.6		
Female	108	70.6	31	70.4	37	69.8	40	71.4		
Race									4.00 (4)	0.41
White	100	65.4	24	54.6	37	69.8	39	69.6		
Black	40	26.1	14	31.8	12	22.6	14	25.0		
Other	13	8.5	6	13.6	4	7.6	3	5.4		
Hispanic ethnicity									1.53 (2)	0.46
No	128	83.7	36	81.8	47	88.7	45	80.4		
Yes	25	16.3	8	18.2	6	11.3	11	19.6		
Education									4.23 (4)	0.38
<12 years	24	15.7	4	9.1	11	20.8	9	16.1		
12–15 years	91	59.5	31	70.4	27	50.9	33	58.9		
>15 years	38	24.8	9	20.5	15	28.3	14	25.0		
Anxious features	114	74.5	30	68.2	42	79.3	42	75.0	1.56 (2)	0.49
Onset of depression before age 18	64	41.8	17	38.6	23	43.4	24	42.9	0.26 (2)	0.88
Continuous variables	Mean	SD	Mean	SD	Mean	SD	Mean	SD	F value (df)	*p* value
Age in years	43.8	11.8	46.8	11.4	45.2	12.0	40.2	11.2	4.64 (2, 150)	0.01
QIDS-SR	15.7	4.0	16.1	3.0	15.1	4.8	16.1	4.0	1.03 (2, 150)	0.36
IDS anhedonia	5.4	2.0	5.3	2.0	5.3	2.1	5.7	1.9	0.76 (2, 150)	0.47
Body mass index	32.0	9.3	33.5	11.5	31.5	7.9	31.2	8.5	0.88 (2, 150)	0.42
Log of S100B	−1.1	1.3	−0.85	1.1	−1.1	1.4	−1.19	1.31	0.88 (2, 150)	0.42

S100B is S100 calcium binding protein B, CO-MED is Combining Medications to Enhance Depression Outcomes, QIDS-SR is Quick Inventory of Depressive Symptomatology Self-Report, IDS is Inventory of Depressive Symptomatology.

**Table 2:** Results of repeated-measures mixed model analyses predicting changes in anhedonia and depression severity based on pre-treatment S100B levels by treatment arms in CO-MED trial.

	Anhedonia Severity			Overall Depression Severity		
	F value	df	*p*	F value	df	*p*
Age	0.32	1, 142	0.57	0.49	1, 142	0.49
Gender	9.71	1, 142	0.002	2.91	1, 142	0.09
Body Mass Index	0.75	1, 142	0.39	0.05	1, 142	0.82
Baseline Log S100B	2.06	1, 142	0.15	0.55	1, 142	0.46
Time	68.18	7, 790	<0.0001	103.58	7, 787	<0.0001
Group	3.08	2, 142	0.049	1.68	2, 142	0.19
Time-by-treatment arm interaction	0.69	14, 790	0.78	0.44	14, 787	0.96
Log S100B-by-treatment arm interaction	3.21	2, 142	0.043	1.99	2, 142	0.14

S100B is S100 calcium binding protein B, CO-MED is Combining Medications to Enhance Depression Outcomes.
